# The 2-Pore Domain Potassium Channel *TREK-1* Regulates Stretch-Induced Detachment of Alveolar Epithelial Cells

**DOI:** 10.1371/journal.pone.0089429

**Published:** 2014-02-19

**Authors:** Esra Roan, Christopher M. Waters, Bin Teng, Manik Ghosh, Andreas Schwingshackl

**Affiliations:** 1 Department of Biomedical Engineering, University of Memphis, Memphis, Tennessee, United States of America; 2 Department of Physiology, University of Tennessee Health Science Center, Memphis, Tennessee, United States of America; 3 Department of Pediatrics, University of Tennessee Health Science Center, Memphis, Tennessee, United States of America; 4 Department of Medicine, University of Tennessee Health Science Center, Memphis, Tennessee, United States of America; Emory University School of Medicine, United States of America

## Abstract

Acute Respiratory Distress Syndrome remains challenging partially because the underlying mechanisms are poorly understood. While inflammation and loss of barrier function are associated with disease progression, our understanding of the biophysical mechanisms associated with ventilator-associated lung injury is incomplete. In this line of thinking, we recently showed that changes in the F-actin content and deformability of AECs lead to cell detachment with mechanical stretch. Elsewhere, we discovered that cytokine secretion and proliferation were regulated in part by the stretch-activated 2-pore domain K^+^ (K2P) channel TREK-1 in alveolar epithelial cells (AECs). As such, the aim of the current study was to determine whether TREK-1 regulated the mechanobiology of AECs through cytoskeletal remodeling and cell detachment. Using a TREK-1-deficient human AEC line (A549), we examined the cytoskeleton by confocal microscopy and quantified differences in the F-actin content. We used nano-indentation with an atomic force microscope to measure the deformability of cells and detachment assays to quantify the level of injury in our monolayers. We found a decrease in F-actin and an increase in deformability in TREK-1 deficient cells compared to control cells. Although total vinculin and focal adhesion kinase (FAK) levels remained unchanged, focal adhesions appeared to be less prominent and phosphorylation of FAK at the Tyr^925^ residue was greater in TREK-1 deficient cells. TREK-1 deficient cells have less F-actin and are more deformable making them more resistant to stretch-induced injury.

## Introduction

Acute Respiratory Distress Syndrome (ARDS) remains a challenging disease to manage in both the adult and pediatric populations [1], [2]. Except for an increased emphasis on low tidal volume ventilation and lung protective strategies, few therapeutic approaches have shown improvement in patient survival [3]–[5]. Oxygen administration and mechanical ventilation, the two main treatment regimens for ARDS, can accentuate lung injury [3], [6], [7]. While inflammation and loss of barrier function contribute to the progression of disease in these patients [8], [9], our understanding of the biophysical mechanisms associated with ventilator-associated lung injury is incomplete [10]–[14].

We recently proposed a regulatory role for the stretch-activated 2-pore domain K^+^ (K2P) channel TREK-1 in the regulation of alveolar epithelial cell (AEC) cytokine secretion and proliferation, two functions related to inflammation and repair [15]–[17]. The main function of K2P channels is thought to be the regulation of the resting cell membrane potential by sustaining so-called “background” or “leak” potassium currents [18]–[20], but these channels may also act as mechanosensors since they are activated by mechanical stretch [21]. Interestingly, in addition to sensing stretch signals, TREK-1 itself has also been shown to mediate changes in intracellular architecture in the anterior eye chamber [22] and the morphology of actin cytoskeleton independent of its channel activity in fetal neurons [23]. Thus, TREK-1 may be involved in both mechanosensing and in regulation of cell structure.

We recently proposed a novel mechanism by which changes in the F-actin content and increased stiffness of AECs could cause loss of barrier function due to stretch-induced cell detachment [24]. A previous study by Yalcin *et al.* [13] demonstrated that actin depolymerization in A549 epithelial cells caused the cells to become softer (more deformable) and protected the cells from injury caused by bubble progression in a model of cyclic collapse and re-opening of airways. Detachment of cells was reduced in softer cells, but paradoxically this correlated with a reduced number of focal adhesions (FAs).

In this study we propose that TREK-1 regulates the deformability of AECs through cytoskeletal remodeling and affects cellular detachment following mechanical stretch. To investigate this hypothesis, we used shRNA transfected, TREK-1-deficient A549 cells, and we show for the first time that TREK-1 deficiency leads to considerable cytoskeletal alterations and an increase in the deformability of human AECs. Moreover, we show that these biophysical changes may be responsible for the decreased cell detachment observed in TREK-1 deficient cells after exposure to mechanical stretch.

## Materials and Methods

### Cell culture

Human A549 AECs were purchased from the American Type Culture Collection (ATCC, Manassas, VA). Cells were cultured in DMEM (Gibco, Carlsbad, CA) supplemented with 10% FBS (Gibco), 1% Penicillin/Streptomycin (Gibco), 20 mM HEPES (Sigma Aldrich, St. Louis, MO), and 2 mM L-Glutamine (Gibco). A stable TREK-1 deficient A549 cell line and a control cell line transfected with a scrambled shRNA were created as previously described [17]. With this approach, we are able to obtain a 63% knock down of TREK-1 expression [15]. Cells were grown on FlexCell plates for detachment, imaging, and biochemical studies, and plastic petri dishes for AFM indentation studies.

### Confocal Microscopy

Confluent cells (80 to 90%) were fixed with 4% paraformaldehyde for 5 min at 4°C, permeabilized with 0.5% Triton X-100 for 10 min, and then blocked with 5% normal horse serum with 2% BSA in PBS for 30 min. The cells were then incubated with Alexa Fluor 594 phalloidin (Life Technologies, Grand Island, NY) for 30 min at room temperature for F-actin staining or an anti-vinculin antibody (Alexa Fluor 488 Vinculin, Millipore) for 2 hours at room temperature. Nuclear staining was obtained using Fluoro Gel II mounting medium containing DAPI (Electron Microscopy Sciences, Hatfield, PA). As a negative control, a species-specific IgG antibody was substituted for the respective primary antibody.

Images were acquired using a Zeiss 710 confocal imaging system. Emitted fluorescence was collected using a 63X magnification objective lens (NA 1.4 Oil), and the images were recorded using Zen 2009 Light Edition software (Zeiss).

### Live cell indentation with atomic force microscopy (AFM)

We utilized the AFM (MFP3D, Asylum Research, Santa Barbara, CA) as a nano-indenter with a flexible cantilever beam (SiNi, Budget Sensors, Sofia, Bulgaria) [24], [25] to measure the elastic modulus, *E*, of live cells. Briefly, the AFM indentation is achieved when the cantilever is lowered enough to come in contact with the cell to cause indentation and deflect the cantilever beam. The reaction force measured on the cantilever beam that compresses the cell is related to the elastic modulus of the cell, the nominal stiffness of the cantilever beam (its spring constant), and the geometry of the tip of the cantilever beam [26]–[28]. The nominal stiffness of the cantilever beams was reported to be 0.27 N/m, but we calibrated the cantilever beam with each experiment. Next, at randomly selected spots on the confluent monolayers, 50 (2×25 grid) force-indentation curves were recorded over a rectangle that is 12.5 µm×50 µm. A MATLAB (Mathworks, Natick, MA) code, developed in our group, was used to batch process the force-indentation curves and to determine the *E* using a modified version of the Hertz equation E  =  F [2(1 – ν^2^)]/[1.4906 δ^2^ tan(θ)] where *ν* is Poisson’s ratio, *θ* is the tip half-opening angle, and *δ* is the sample indentation [26]–[28]. In the analyses, the Poisson’s ratio is assumed to be 0.49. We obtained a minimum of 10 maps from 2 petri dishes per condition from 4 different cell-seeding events. We computed the median modulus of each map and averaged these over the dish.

### Cell Detachment Experiments and Quantification

Confluent monolayers of A549 cells were exposed to cyclic stretch using the Flexercell FX-4000T tension unit (Flexcell International, Hillsborough, NC). Cells were exposed to 20% linear strain for 8 hours at a frequency of 15 cycles/min and fixed in 4% paraformaldehyde for 5 min at 4°C. This is considered to be an injurious level of stretch considering that AECs in the lungs experience ∼4% stretch during normal tidal breathing [29], [30]. Phase contrast images were collected at 20X magnification using an EVOS digital microscope (5 images/well) and marked using Adobe Photoshop CS6. Images were analyzed using MATLAB (Natick, MA) to determine the percentage of denuded area relative to the overall field. Unstretched cells were used as controls.

### Western Blot Analysis

**G- and F-actin assay.** Cells were seeded in 6 well FlexCell plates (0.3×10^6^ cells/well) in triplicate and grown to >90% confluence. Cells were washed twice with cold PBS and then lysed in the following solution for 5 min on ice: 1% Triton X-100, 20 mM Tris, 5 mM EGTA, 20 mM NaFl, 25 mM Na pyrophosphate, containing a protease inhibitor cocktail (Roche, Burlington, NC). G-actin containing supernatants were collected and total protein concentrations were determined using the Quick Start Bradford (BioRad, Hercules, CA). Thereafter, F-actin was extracted by adding the following solution: 1% Triton X-100, 20 mM Tris, 5 mM EGTA, 20 mM NaFl, 25 mM Na pyrophosphate, containing a protease inhibitor cocktail (Roche, Burlington, NC) and 5% SDS and 5% deoxycholic acid. After 5 min, F-actin was removed from the wells using a cell scraper and the samples were centrifuged at 17,000 g for 20 min at 4°C. To determine the relative amounts of G- and F-actin, we performed Western Blot experiments loading equal amounts of volume per lane with an antibody against actin (1:1000, Cytoskeleton, Denver, CO). GAPDH (1:2000, Cell Signaling) was used as an internal loading control for G-actin.

**Phospho-FAK, total FAK, and total vinculin determination.** To determine the amounts of vinculin, focal adhesion kinase (FAK), phospho FAK (Tyr^397^), and phospho FAK (Tyr^925^) in our samples, we seeded 0.3×10^6^ cells in 6 well plates. Once cells reached 90% confluence, they were lysed on ice in RIPA buffer (50 mM Tris·HCl, pH 7.4, 150 mM NaCl, 2 mM EDTA, 1% Nonidet P-40, 0.1% SDS) with a protease inhibitor cocktail (Roche, Burlington, NC). Lysates were centrifuged at 4°C and 17,000 g for 15 min, and total protein concentrations were measured using the Bradford assay (BioRad, Hercules, CA), with 60 µg protein loaded in each lane. An antibody pair against phospho FAK (Tyr^397^, 1∶1000 dilution), total FAK (1∶1000 dilution), and vinculin (1∶1000 dilution) was purchased from Cell Signaling, and another antibody pair against phospho FAK (Tyr^925^; 1∶1000 dilution) and total FAK was purchased from Abcam (Cambridge, MA). GAPDH (1∶2000) was used as an internal loading control.

**Gel electrophoresis.** G-actin, F-actin, phospho FAK (Tyr^397^ and Tyr^925^), and total FAK samples were separated by sodium dodecyl sulfate (SDS)- polyacrylamide gel electrophoresis (PAGE) on 4–12% NuPage Bis-Tris gradient gels (Life Technologies, Grand Island, NY) and transferred onto nitrocellulose membranes at 35 mV for 2 hours. All membranes were blocked in 5% non-fat dry milk in Tris-buffered saline (Bio-Rad) containing 0.1% Tween-20 for 1 h at 37°C. The membranes were then incubated overnight with the indicated primary antibodies at 4°C. The next day, all membranes were incubated for 1 hour with an anti-rabbit HRP-conjugated IgG (1∶2000, Cell Signaling). To detect GAPDH we used an anti-rabbit HRP-conjugated IgG (1∶5000, Cell Signaling). Bands were visualized by enhanced chemiluminescence with ECL SuperSignal West Dura Extended Duration Substrate (Thermo Scientific, Rockford IL). Band densitometry to determine relative quantities of protein were performed using ImageJ 1.42 software for Windows.

### Statistical analysis

All values were expressed as mean ± SEM and statistical analysis was performed using Student’s t-test or ANOVA. All statistical analyses were performed using SigmaStat 3.5 software, and a p-value of p<0.05 was considered significant.

## Results

### TREK-1 deficient cells contained less F-actin

**Figure 1:**
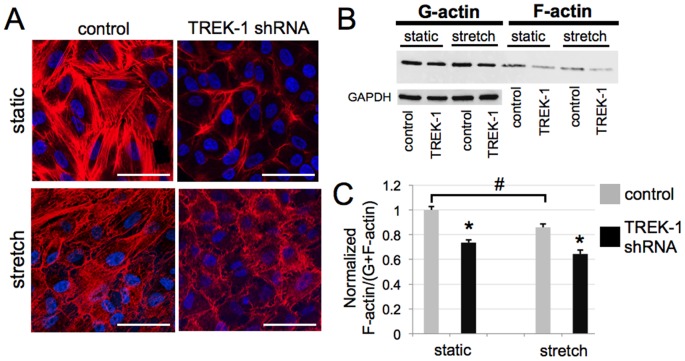
The normalized ratio of F-actin to total (G+F) actin in TREK-1 deficient cells is decreased compared to control cells. (A) Representative confocal microscopy images of 3 separate experiments demonstrating decreased F-actin staining (phalloidin, red) in TREK-1 deficient A549 cells compared with control shRNA cells under both no stretch (static) and stretch conditions. Nuclei were counterstained with DAPI (blue). (B) Representative Western blots showing a decrease in the F-actin content relative to G-actin in TREK-1 deficient A549 cells. GAPDH was used as an internal loading control for the G-actin (Triton-soluble) fraction. (C) Densitometric analysis shows a significant decrease in the ratio of F-actin relative to total (G+F) actin. Values were normalized to the ratio in unstretched control cells. Cyclic stretch was applied for 8 hr (15 cycles/min, 20%). (n = 4,*p<0.05, compared to unstretched control cells; # unstretched control cells compared to stretched control cells).

To test the hypothesis that TREK-1 regulates cell structure and deformability, we compared the distribution of F-actin and the relative content of F- and G-actin in control and TREK-1-deficient AECs. Using confocal fluorescence microscopy, we found that TREK-1-deficient A549 cells exhibited decreased F-actin compared with control cells (Figure 1A). An extensive network of F-actin stress fibers was observed in the unstretched (static) control cells, while stress fibers were much less prominent in the TREK-1-deficient cells. In contrast, we found no difference in the appearance of α-tubulin immunofluorescence between control and TREK-1 deficient cells (results not shown).

To determine whether the decrease in F-actin stress fibers in TREK-1 deficient cells was due to changes in actin content, we examined the content of Triton-soluble (mainly G-actin) and Triton-insoluble (mainly F-actin) fractions. The representative Western blots in Fig. 1B show that both cell types contained similar amounts of G-actin, but TREK-1-deficient cells contained decreased amounts of F-actin compared to control cells (Fig. 1B). Band densitometry analysis shown in Fig. 1C demonstrates that TREK-1-deficient cells contained significantly less (27.2%) F-actin relative to total (G+F) actin compared with control cells.

To further investigate the effect of TREK-1 deficiency on the cell cytoskeleton, we exposed cells to cyclic stretch (20% strain, 15 cycles/min) for 8 hr. We found that cyclic stretch altered the distribution and appearance of F-actin (Fig. 1A) as well as the relative amount of F-actin in control shRNA cells (Fig. 1C). Cyclic stretch caused F-actin to appear as stress fibers that had been elongated and then released. In TREK-1-deficient cells, cyclic stretch induced a similar appearance of F-actin (Fig. 1A), but the ratio of F-actin to total actin was unaffected (Fig. 1C).

### TREK-1 deficiency increased the deformability of AECs

**Figure 2:**
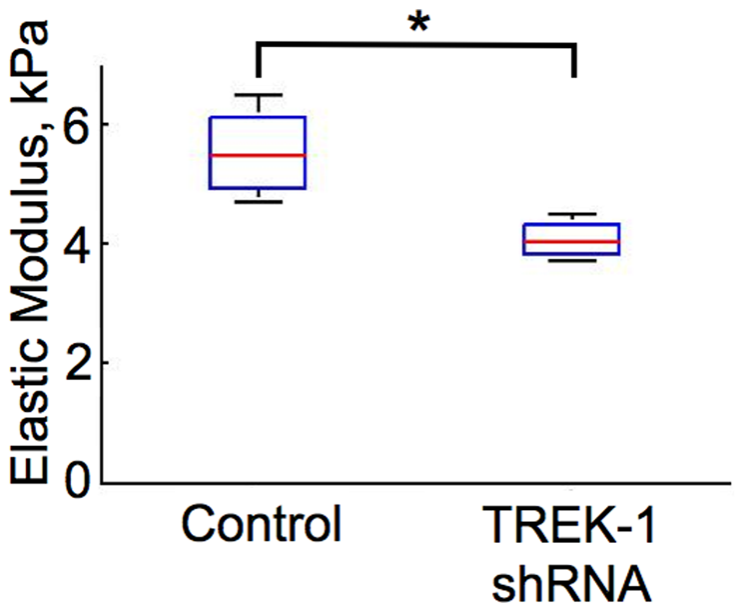
TREK-1 deficiency increased the deformability of cells. TREK-1 deficient cells have a significantly lower elastic modulus (E) than control cells. AFM indentation was conducted on live monolayers of A549 cells as described in the methods. (n = 4, *p<0.05.)

To determine whether the observed changes in the actin content of TREK-1 deficient cells impacted the deformability of live AECs, we used nano-indentation to measure the elastic modulus (E); the lower the E, the more deformable the cells. As seen in Fig. 2, the average E of TREK-1 deficient cells was significantly lower than in control cells. The E of control cells was 5.47±0.45 kPa whereas the E of TREK-1 deficient cells was 4.03±0.19 kPa (p<0.05, n = 4). This is consistent with the finding that TREK-1 deficient cells contained less F-actin.

### TREK-1 deficiency protected AECs from detachment caused by cyclic stretch

**Figure 3:**
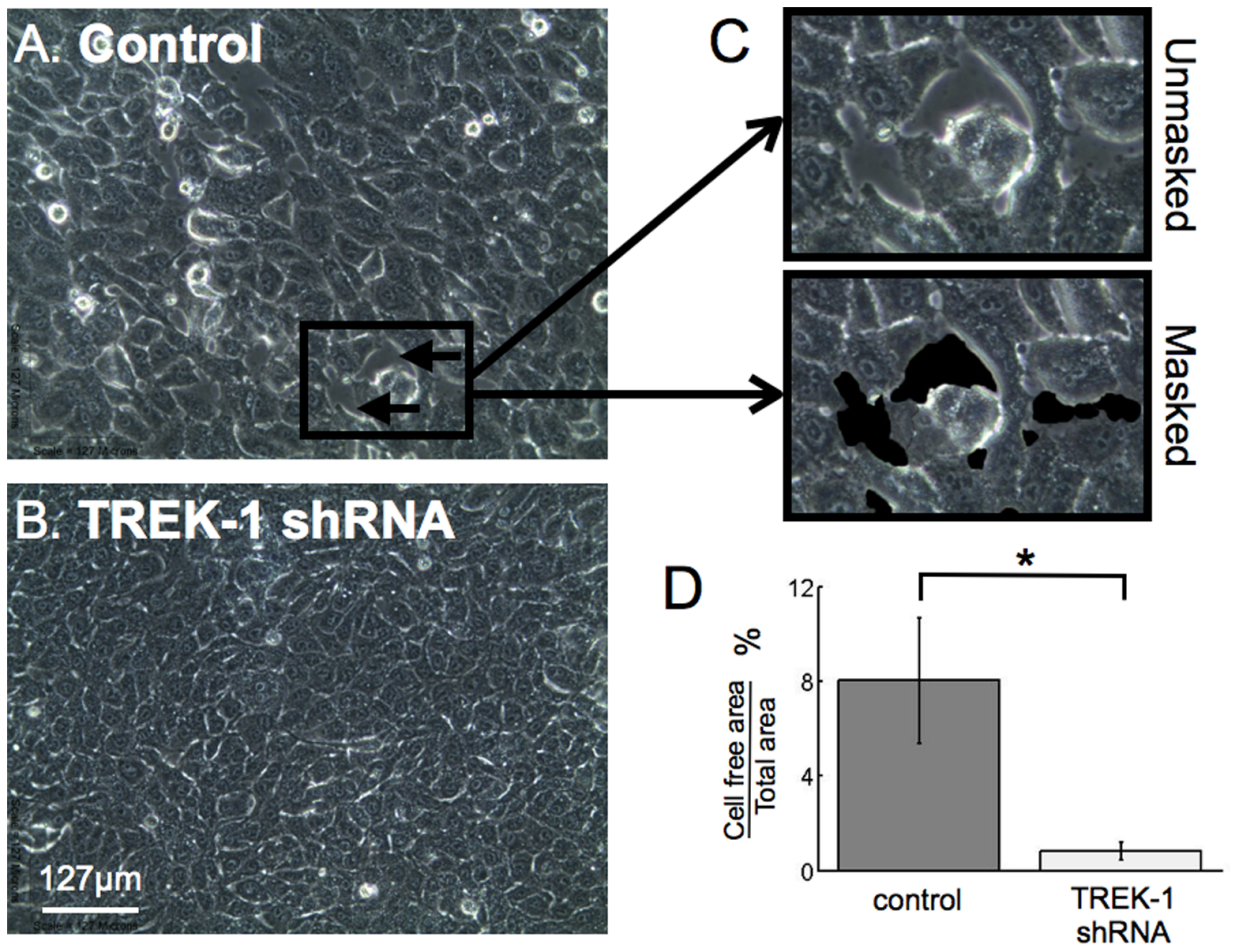
TREK-1-deficient cells were protected from stretch-induced cell detachment. Representative images of control (A) and TREK-1-deficient (B) A549 cells after stretch. (C) A close up of Panel A where an area with cell denudation is captured and masked (black areas). (D) Summary of 4 separate experiments indicating a significant decrease in cell detachment in TREK-1-deficient cells (5 images per well, 3 wells per cell type in each experiment). (n = 4, *p<0.05.)

In a previous study, we showed that cells with a higher elastic modulus (less deformable) were more susceptible to stretch-induced cell detachment. To determine whether TREK-1 deficiency impacted cell detachment caused by stretch, we quantified cell-free area after 8 hours of cyclic stretch exposure. As shown in Fig. 3, we found 10 times more cell detachment (cell-free area) in control cells compared to TREK-1 deficient cells (8.0%±2.6%(STE) vs. 0.8%±0.4%(STE)). These results suggest that TREK-1 deficiency may protect the alveolar epithelial barrier from stretch-induced cell detachment.

### TREK-1 deficiency caused changes in the distribution of vinculin and phosphorylation of FAK

**Figure 4:**
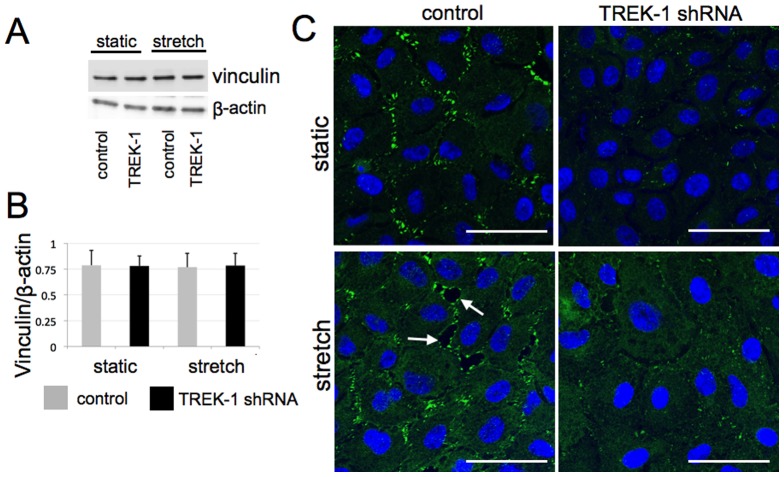
Vinculin expression was not affected by TREK-1 deficiency, but localization to focal adhesions was diminished. (A) Representative Western Blot experiment depicting total vinculin expression under no stretch (NS) and stretched (stretch) conditions. (B) Densitometric quantification of 3 experiments showing equal levels of total vinculin expression in control and Trek-1 deficient cells under no stretch (static) and stretch conditions. (C) Representative confocal microscopy images of vinculin immunofluorescence (green) and nuclei (DAPI, blue) in unstretched cells (static) and stretched cells (20% stretch, 15 cycles/min) for 8 hr; n = 3. All cells were grown on FlexCell plates.

Focal adhesions (FAs) are located at the interface between cells and extracellular matrix and play a key role in the regulation of the biophysical properties of a cell. Vinculin is a membrane-cytoskeletal protein in FAs that is involved in the linkage of integrin adhesion molecules to the actin cytoskeleton and participates in the regulation of AEC adherence to submucosal substrates [31]. To determine whether the biophysical changes induced by TREK-1 deficiency or cyclic stretch altered FAs, we examined vinculin expression by western blotting and vinculin organization by immunofluorescence microscopy (Fig. 4). The total vinculin content was similar between control and TREK-1 deficient cells, and was not altered by cyclic stretch (Fig. 4A-B). As shown in Fig. 4C, unstretched (static) control cells exhibited a distinct vinculin staining pattern indicative of FAs, but these structures were not prominent in TREK-1-deficient cells. When cyclic stretch was applied, vinculin staining appeared more diffuse and FAs localized near gaps between cells in control cells. TREK-1-deficient cells also exhibited more diffuse vinculin staining but no change in FAs.

**Figure 5:**
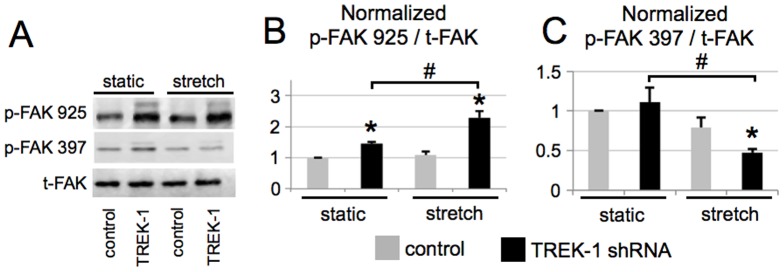
Total FAK was unchanged by TREK-1 deficiency, but FAK phosphorylation at Tyr^925^ residue was increased in TREK-1 deficient cells, while Tyr^397^ phosphorylation was decreased in stretched cells. (A) Representative Western blots of FAK Tyr^397^ and Tyr^925^ phosphorylation in control and TREK-1 deficient cells under no stretch (static) and stretched (20%, 15 cycles/min for 8 hours) conditions. (B) Summary of densitometric band analyses of 3 Western blot experiments for Tyr^925^ and for Tyr^397^ (C). In (B) and (C) the ratio of phosphorylated FAK (p-FAK) to total FAK (t-FAK) was normalized to the ratio for unstretched control cells. *p<0.05, compared to unstretched control cells; # unstretched TREK-1 deficient control cells compared to stretched TREK-1 deficient cells; n = 3-4.

Phosphorylation of focal adhesion kinase (FAK) at specific residues can indicate activation of FAK, which may indicate participation in the formation or disorganization of FAs [32]. We found that total FAK expression was similar in control and TREK-1 deficient cells and was not affected by cyclic stretch (Fig. 5). However, phosphorylation of FAK at the Tyr^925^ residue was increased in TREK-1 deficient cells, and was further increased in cells exposed to cyclic stretch. In contrast, phosphorylation of FAK at the Tyr^397^ site was not affected by TREK-1 deficiency in unstretched cells, but was significantly decreased in TREK-1 deficient cells after stretch (Fig. 5).

## Discussion

In a previous study, we demonstrated that loss of AEC deformability increased cell detachment due to stretch [24]. Based upon these findings and a report proposing that TREK-1 expression could alter cytoskeletal remodeling [23], we hypothesized that TREK-1 was involved in the regulation of cell deformability. In the present study, we showed that decreased expression of TREK-1 in A549 cells caused changes in the cytoskeletal structure, localization of FAs, and an increase in cell deformability. These changes provided protection against stretch-induced cell detachment. Interestingly, in our previous study, we showed that a decrease in cell deformability (stiffer cells) led to increased cell detachment [24]. In this study, we further expand this concept by showing that an increase in cell deformability (softer cells) led to decreased cell detachment when exposed to cyclic stretch. In our studies, we have utilized an injurious level of linear strain of 20% based on our prior experience [24], [33]. In future studies, we intend to explore the role of stretch frequency (strain rate), magnitude, and duration on cell detachment of control and TREK-1 deficient cells.

It is an interesting finding that A549 cells with a more deformable cytoskeleton and less organized FAs display less injury or better attachment to a substrate experiencing stretch. This suggests that cells with greater deformability (less cytoskeletal stress) and decreased stress at sites of FAs are better able to withstand deforming stresses. These findings are consistent with the earlier findings of Yalcin *et al.* in which they demonstrated that AEC injury and detachment due to bubble propagation across the cells was decreased in cells with de-polymerized actin [13]. Interestingly, they also observed that the protected cells were softer and exhibited reduced FAs. We previously showed that disruption of F-actin with cytochalasin D caused a significant reduction in cell deformability [24]. One limitation in our study is that the elastic moduli of the substrates (1-2MPa for FlexCell membranes, ∼2GPa for petri dishes) are significantly greater than the lung parenchyma (∼5 kPa)[34], [35]. There is now extensive information relating various functions of cells (e.g., fibrotic activity [36]) and substrate stiffness, although not all cell types exhibit the same level or type of sensitivity [37]. Without further experiments with cells grown on substrates with very low E (<100 kPa) that can be mechanically stretched, we cannot predict whether differences we observed between control and TREK-1 shRNA cells would be altered.

TREK-1 deficient cells had less detachment in response to stretch even though they contained less organized FAs (Fig. 4). We then examined if the FA alterations in TREK-1 deficient cells were associated with FAK phosphorylation. The relationship between phosphorylation of FAK at different residues, the maturation or turnover of FAs, and cell attachment is complex and not fully understood [38]. Previous studies have shown that FAK was phosphorylated at Tyr^397^ and Tyr^925^ residues at the onset of migration in A549 cells [39] or in mouse embryonic fibroblasts [40]. It is possible that FAK is phosphorylated to disassociate from FAs to encourage turnover, thus facilitating migration. We found that the baseline level of FAK phosphorylation was higher in TREK-1 deficient cells at the Tyr^925^ residue (Fig. 5B), which is known to lead to disorganization and turnover of FAs [38], [41]. This may explain the less prominent formation of FAs in our unstretcthed TREK-1 deficient cells (Fig. 4C). Although we provide strong evidence that TREK-1 appears to modulate the cell cytoskeleton, the molecular mechanisms by which TREK-1 alters the cell cytoskeleton remain elusive.

In summary, for the first time, we demonstrated that reduced expression of the stretch-activated K2P channel TREK-1 in A549 cells caused a decrease in F-actin content, an increase in cell deformability, reduced localization of vinculin at FAs, and increased phosphorylation of FAK at Tyr^925^. These changes promoted protection of the cells against cell detachment caused by cyclic stretch. The reduction in E and F-actin content suggest a more deformable cytoskeleton that is capable of sustaining injurious stretch levels. However, the molecular mechanisms linking TREK-1 to changes in actin/vinculin/FAK remain to be determined. It is quite possible that TREK-1 may function not only as a K^+^ permeable pore, but also a regulatory molecule, as has been described for the Cl^−^ permeable channel CFTR [42]. In fact, Lauritzen *et al.* [23] proposed a direct regulatory role of TREK-1 on cytoskeletal remodeling independent of its function as an ion channel. Further studies are necessary to delineate the molecular details of such a relationship.
